# Effect of sea buckthorn extract on production performance, serum biochemical indexes, egg quality, and cholesterol deposition of laying ducks

**DOI:** 10.3389/fvets.2023.1127117

**Published:** 2023-02-27

**Authors:** Bing-nong Yao, Fu-you Liao, Jiao-yi Yang, Ai Liu, Jiao Wang, Bao-guo Zhu, Gang Feng, Sheng-lin Yang

**Affiliations:** ^1^Key Laboratory of Animal Genetics, Breeding and Reproduction in the Plateau Mountainous Region, Ministry of Education, College of Animal Science, Guizhou University, Guiyang, China; ^2^College of Animal Science, Guizhou University, Guiyang, China

**Keywords:** sea buckthorn extract, laying duck, production performance, egg quality, amino acid, fatty acid, serum biochemical indices, cholesterol deposition

## Abstract

The purpose of this experiment was to study the effect of sea buckthorn extract (SBE) supplementation on the production performance, serum biochemical indexes, egg quality, and cholesterol deposition of laying ducks. A total of 240 23-week-old laying ducks (female ducks) with similar body weight were randomly divided into four treatment groups with 6 replicates of 10 each. The experimental groups were fed diets supplemented with 0, 0.5, 1.0, and 1.5 g/kg of SBE, respectively. The results showed that the addition of 1.0 g/kg SBE to the diet had significant increase (*P* < 0.05) in average egg weight and feed conversion ratio. The inclusion of SBE showed the significant improvement (*P* < 0.05) in yolk weight, shell strength, egg white height and haugh unit. Ducks fed with 1.0 and 1.5 g/kg SBE displayed a significant decrease (*P* < 0.05) in yolk cholesterol. The significant improvements were observed in the contents of total amino acid essential amino acids, non-essential amino acids, umami amino acids, monounsaturated fatty acids, and docosahexenoic acids of eggs (*P* < 0.05) when supplemented with SBE. However, the contents of total saturated fatty acids, polyunsaturated fatty acids, n-3 polyunsaturated fatty acids and n-6 polyunsaturated fatty acids in eggs showed decrease when ducks fed with SBE diets (*P* < 0.05). SBE diets may reduce (*P* < 0.05) the levels of serum total cholesterol, triglyceride, and low-density lipoprotein cholesterol, while increased (*P* < 0.05) the levels of serum superoxide dismutase, total antioxidant capacity, and glutathione catalase compared to the control. The levels of serum immunoglobulin G, immunoglobulin A and immunoglobulin M were improved in SBE diets (*P* < 0.05) in comparation to the control. The addition of SBE to diets can improve feed nutrient utilization, increase egg weight, optimaze egg quality and amino acid content in eggs, reduce blood lipids, improve fatty acid profile and yolk cholesterol in eggs, and increase antioxidant capacity and immunity in laying ducks.

## 1. Introduction

Meat and egg products from poultry serve as a source of food for humans, especially eggs can supply humans with high quality proteins, of which eggs are rich in many umami amino acids such as glutamic acid and glycine (1). Fatty acids in eggs are also beneficial for human health, among which n-3 polyunsaturated fatty acids in eggs are effective in preventing cardiovascular diseases (2), but the docosahexaenoic acid (DHA) content of n-3 polyunsaturated fatty acids in poultry meat and eggs is known to be very low (3). Fat and cholesterol enriched in eggs are mainly synthesized in the liver and transported *via* the blood to be deposited in eggs (4, 5). Generally, consumption of one to two eggs per day is sufficient to meet the body's dietary cholesterol requirements, and excessive intake can cause cardiovascular disease. Therefore, how to balance the nutritional structure in eggs has become a hot research topic. On the other hand, the use of antibiotics has led to the detection of antibiotic residues in a large number of livestock and poultry products, posing a safety risk to human health (6). Therefore, countries have introduced a series of regulations for the use of antibiotics, and China has explicitly prohibited the use of antibiotics in feed, and it has become a trend to find alternative antibiotic products from feed additives. It has been reported that plants and plant extracts can be used as feed additives to improve egg production performance, egg quality and immunity of chickens, and to reduce cholesterol levels (7). As everyone knows, plants are rich in many natural bioactive compounds, such as flavonoids and polysaccharides, among which mulberry leaf flavonoids can improve egg production and antioxidant capacity of chickens and improve feed conversion ratio (8).

Sea buckthorn (*Hippophae rhamnoides L*., SBT) for the elaeagnaceae family, the genus of sea buckthorn. SBT is cold and drought tolerant and can grow in harsh environments. SBT is abundant and native to China and is distributed all over the world, including England and France (9). Feeding of SBT to broiler diets can improve feed conversion (10). SBT is a medicinal and food plant, and the flavonoids, polysaccharides, and other bioactive compounds extracted from the stems, leaves, fruits, and seeds of SBT are collectively known as Sea buckthorn extract (SBE) (11). Among them, Seabuckthorn flavonoids (SBF) has the largest proportion and is the most abundant active compound in SBT. SBE has high medicinal value and the medicinal components are isorhamnetin, quercetin, and kaempferol (12), and its medicinal functions are lipid-lowering (cholesterol, triglycerides, etc.) (13), antioxidant (14), anticancer, and immunomodulation (15), etc. It has been reported that SBT and SBE were widely used as feed additives in pig (16), cattle (17), chicken (18), and other production tests. To our knowledge, few studies on effect of SBE in ducks have been reported, especially, little is known about the study of amino acids, fatty acid profile, and egg quality in duck eggs.

Guangxi small hemp duck is a local Chinese poultry breed, produced in Guangxi, China, and is an egg-laying duck with good egg production performance. However, duck eggs contain higher levels of cholesterol and fat than chicken eggs and quail eggs, and humans are concerned about the health risks associated with high fat and cholesterol levels when consuming them. Therefore, we hypothesized that the addition of SBE to the diet could improve the production performance of laying ducks, improve egg quality, improve the amino acid and fatty acid structure of eggs, and reduce egg cholesterol. The aim of this study was to investigate the effect of SBE on production performance, serum biochemical parameters, egg quality, amino acid, fatty acid profile of egg, and cholesterol deposition during the laying period, and to provide a theoretical basis for the development of SBE as a functional feed additive for laying ducks.

## 2. Materials and methods

This experiment was reviewed and approved by the Guizhou University Sub-Committee of Experimental Animal Ethics (Guiyang, China; No. EAE-GZU-2021-E012).

### 2.1. Experiment material

The raw material of sea buckthorn extract (powdered form) used in the experiment was sea buckthorn fruit, which was produced in Shaanxi, China. The active ingredient of SBE was detected by high performance liquid chromatography-mass spectrometry, and the extractant was alcohol. 286.23 mg/g of flavonoids, 103.79 mg/g of quercetin, and 23.66 mg/g of kaempferol constituted the main components of SBE, and the purity of SBE was 40.31%. Another 20.18% was sea buckthorn polysaccharide. Produced by a company in Shanxi, China.

### 2.2. Experimental design

This experiment was carried out in the research duck farm of Guizhou University from September 2021 to December 2021. The experimental ducks, called Guangxi small hemp duck, were purchased from a farm in Guangxi, China, A total of two hundred and forty 23-week-old laying ducks (female ducks) with similar body weight (BW, 1,250 ± 60 g; mean ± standard deviation) were applied and randomly divided into four groups, every group contained six replicates of 10 ducks each. The experimental diets were supplemented with 0.5, 1.0, and 1.5 g/kg SBE, respectively. The basal rations were formulated according to the Criterion of Nutrients Requirements of laying ducks (SAC, GBT/41189-2021; China, 2021). The basal diet formula and nutritional level are shown in Table 1. Nutrients in the diets were tested using the AOAC method (19). The adaptation period of this experiment was 14 days, and the formal experimental period was 70 days. During the experiment, the ducks were provided with natural and artificial lighting to ensure 16 h of light each day, and the relative humidity was kept at about 65%. Feeding and drinking water freely throughout the feeding period, and the duck house was cleaned once a day.

**Table 1:** Basic diet formula and nutrition level.

**Ingredients, %**	**Content, %**
Corn	55.75
Soybean meal	27.40
Wheat bran	1.50
Rapeseed cake	4.00
CaHPO_4_	2.75
Limestone	7.25
NaCl	0.35
Premix^a^	1.0
Total	100
**Nutrient levels** ^b^	
Metabo lizable energy, MJ/kg	10.65
Crude protein	18.10
Crude fiber	3.07
Calcium	3.37
Total phosphorus	0.63
Lysine	0.92
Methionine	0.27
Methionine + cysteine	0.61

a The premix provided the following per kg of diets: retinol 4,000 IU; oryzanin 1.17 IU; pyridoxol 3.05 IU; cobalamin 0.01 IU; cholecalciferol 900 IU; tocopherol 20 IU; menadione 2 mg; biotin 0.1 mg; folic acid 1.0 mg; pantothenic acid 10 mg; nicotinic acid 50 mg; Cu 10 mg as copper sulfate; Fe 80 mg as ferrous sulfate; Mn 60 mg as manganese sulfate; Zn 60 mg as zinc sulfate; I 0.40 mg as potassium iodide; Se 0.20 mg as sodium selenite.

b Crude protein and crude fiber are measured values, while the others were calculated values.

### 2.3. Sample collection and index determination

#### 2.3.1. Production performance

The daily feed intake was recorded according to replicates during the experiment. The ducks were fed at 7:30 and 17:00 daily, and duck eggs were collected every morning before feeding, numbered and weighed. The egg-laying ducks were weighed every Sunday at 8:00 (fasting ducks for 12 h before weighing). At the same time, the number of deaths of laying ducks was recorded for calculating the average daily feed intake [ADFI (g/d) = cumulative feed intake/(number of birds × number of days)], average egg weight (AEW = total daily egg mass/laying number), laying rate [LR (%) = (laying number/layer number) × 100], and feed conversion ratio [FCR = total feed intake/total egg weight] of the laying ducks. Used to calculate the production performance of egg ducks.

#### 2.3.2. Serum biochemical indices

On day 70 of the experiment, 2 laying ducks with similar body condition were randomly selected from each replicate (12 laying ducks in each group) for blood collection, and the ducks drank freely and fasted for 12 h before blood collection. The blood samples were collected from the vein under the wing of common collecting blood vessel for 5 mL, left at room temperature for 2 h, centrifuged at 1,487 xg/min for 15 min, and the serum was extracted, stored at −80°C for determination of serum biochemical parameters. Serum biochemical indicators include total cholesterol (TC, COD-PAP method), triglyceride (TG, GPO-PAP enzymatic method), high-density lipoprotein cholesterol (HDL-C, direct method), low-density lipoprotein cholesterol (LDL-C, direct method), glutathione catalase (GSH-Px, colorimetric method), total superoxide dismutase (SOD, extraction method), total antioxidant capacity (T-AOC, ratio of Color method), malondialdehyde (MDA, TBA method). Determination of duck serum immunoglobulin G (IgG, Elisa method), immunoglobulin A (IgA, Elisa method), and immunoglobulin M (IgM, Elisa method) using Elisa kits. The kit was sourced from Nanjing Jiancheng Bioengineering Research Institute Co., Ltd., China, and the assay methods and steps were operated in accordance with the kit instructions. The detection instrument used in the experiment was PowerWaveXS type full-wavelength microplate reader (Bio-tek Instruments.Inc.USA).

#### 2.3.3. Egg quality index

On the 69th day of the experiment, three fresh duck eggs with similar morphology were randomly selected in each replicate for egg quality determination (18 eggs per group), the following indicators were measured according to the method of “Performance ferms and measurement for poultry” (NY/T 823-2004) (20). The measuring instruments are listed as follows: Egg weight (DJ-A1000 electronic balance, Connecticut HZ Electronics Co., Ltd., USA), egg shell strength (EFA-01 egg shell strength tester, Orka, Israel), egg shell thickness (MNT-150T digital Vernier caliper, Shanghai Minette Industrial Co., Ltd., China), egg shape index (calculated by vernier caliper), egg yolk specific gravity (calculated by electronic balance weighing), egg white height (EA-01 egg quality tester, Orka, Israel), egg yolk Color (EA-01 egg quality tester, Orka, Israel), Haugh Units (EA-01 egg quality tester, Orka, Israel).

Three fresh duck eggs were randomly selected from each replicate for the determination of yolk cholesterol on the 69th day of the experiment. First, break the fresh duck egg to separate the yolk, weigh 1 g of egg yolk in the middle of the egg yolk and place it in a 10 mL centrifuge tube, add 10 mL of anhydrous ethanol, and mix thoroughly to obtain the sample to be tested, which is used to determine the total protein and total cholesterol of the egg yolk, and then the cholesterol content of the egg yolk was calculated using the formula. Determination formula: Yolk cholesterol (mmol/gprot) = (A_sample_-A_blank_)/(A_calibration_-A_blank_)×C_calibrators_×C_standard_/(W/V), (A_sample_, sample OD value; A_calibration_, calibrate the OD value; A_blank_, blank OD value; C_calibrators_, calibrator concentration, mmol/L; C_standard_, protein concentration of the sample to be tested, gprot/L; W, sample quality, g; V, the total volume of ethanol added, L; prot, protein.). Wavelengths of 570 and 440 nm.

#### 2.3.4. Detection of amino acids and fatty acids in eggs

On the 70th day of the experiment, three fresh duck eggs were randomly selected from each replicate (18 duck eggs in each group, fully mixed, and three samples taken for testing). At first, the fresh duck eggs were dried in a vacuum freeze-dryer (LYOQUEST-85PLUS, Telstar Electromechanical Equipment Shanghai Co., Ltd., China) until Constant weight was reached, secondly, the egg powder samples were obtained by grinding with high-speed multi-function grinder (model 800Y, Wuyi County Hainer Electric Co., Ltd., China).

Amino acids in whole eggs were analyzed according to the national standard for food safety GB/T5009.124-2016 (21). The brief steps are as follows: add 10–15 mL 6 mol/L hydrochloric acid solution and 4 drops of phenol into the hydrolysis tube, after freezing the hydrolysis tube for 5 min and then connected to the suction tube of the vacuum pump, evacuated (close to 0 Pa), filled with nitrogen gas. The vacuum-nitrogen filling step was repeated three times. The sealed tube was hydrolyzed in a 110°C hydrolysis furnace for 22 h, then cooled to room temperature and detected by an automatic amino acid analyzer (Biochrom 30, Biochrom Ltd., UK) at wavelengths of 570 and 440 nm. The 16 amino acids are as follows: aspartic acid (Asp), threonine (Thr), serine (Ser), glutamic acid (Glu), glycine (Gly), alanine (Ala), valine (Val), methionine (Met), isoleucine (Ile), leucine (Leu), tyrosine (Tyr), phenylalanine (Phe), histidine (His), Lysine (Lys), arginine (Arg), and proline (Pro). All experimental steps were completed in strict accordance with the standard instructions, and the difference of the measurement results did not exceed 12% of the arithmetic mean.

The fatty acids in whole eggs were analyzed according to GB5009.168-2016 (22), the national standard for food safety. Brief steps are as follows: weigh 2 g of sample into a 50 mL test tube, add 20 mL of chloroform and 10 mL of methanol, sonicate for 10 min, and shake for 2 h. Add 6 mL of 0.9% sodium chloride aqueous solution, shake for 30 s, and let stand at 4°C for 22 h, then centrifuge at 3,500 r/min for 10 min, collect the lower chloroform layer solution, filter with filter paper. The filtrate was placed in a dry flask and dried in a vacuum drying oven. The single fatty acid methyl ester standard solution and the fatty acid methyl ester mixed standard solution were injected into the gas chromatograph separately to characterize the peaks. The individual fatty acids were analyzed by gas chromatograph (Agilent GC 6890N, Agilent, USA). The gas chromatographic conditions were as follows: the capillary column was a poly (dicyanopropyl siloxane) strongly polar stationary phase (100 m × 0.25 mm × 0.2 μm). The injector temperature was set at 270°C; the detector temperature was set at 280°C. The initial temperature program was 100°C for 13 min, 10°C/min to 180°C, duration 6 min, 1°C/min to 200°C, duration 6 min, and 4°C/min to 230°C, duration 6 min. The carrier gas is nitrogen, the split ratio was 100:1, and the injection volume was 1.0 μL. All experimental steps were performed in strict accordance with the standard requirements, and the difference of the measurement results did not exceed 10% of the arithmetic mean.

### 2.4. Statistical analysis

All data were analyzed by analysis of variance using the general linear model program of SPSS 25 (One-way ANOVA, LSD), and Duncan's multiple comparison test was used. A *p*-value of <0.05 was considered statistically significant. The experimental results of each group were expressed using the mean and standard error of the mean (SEM).

## 3. Results

### 3.1. Production performance

**Figure 1:**
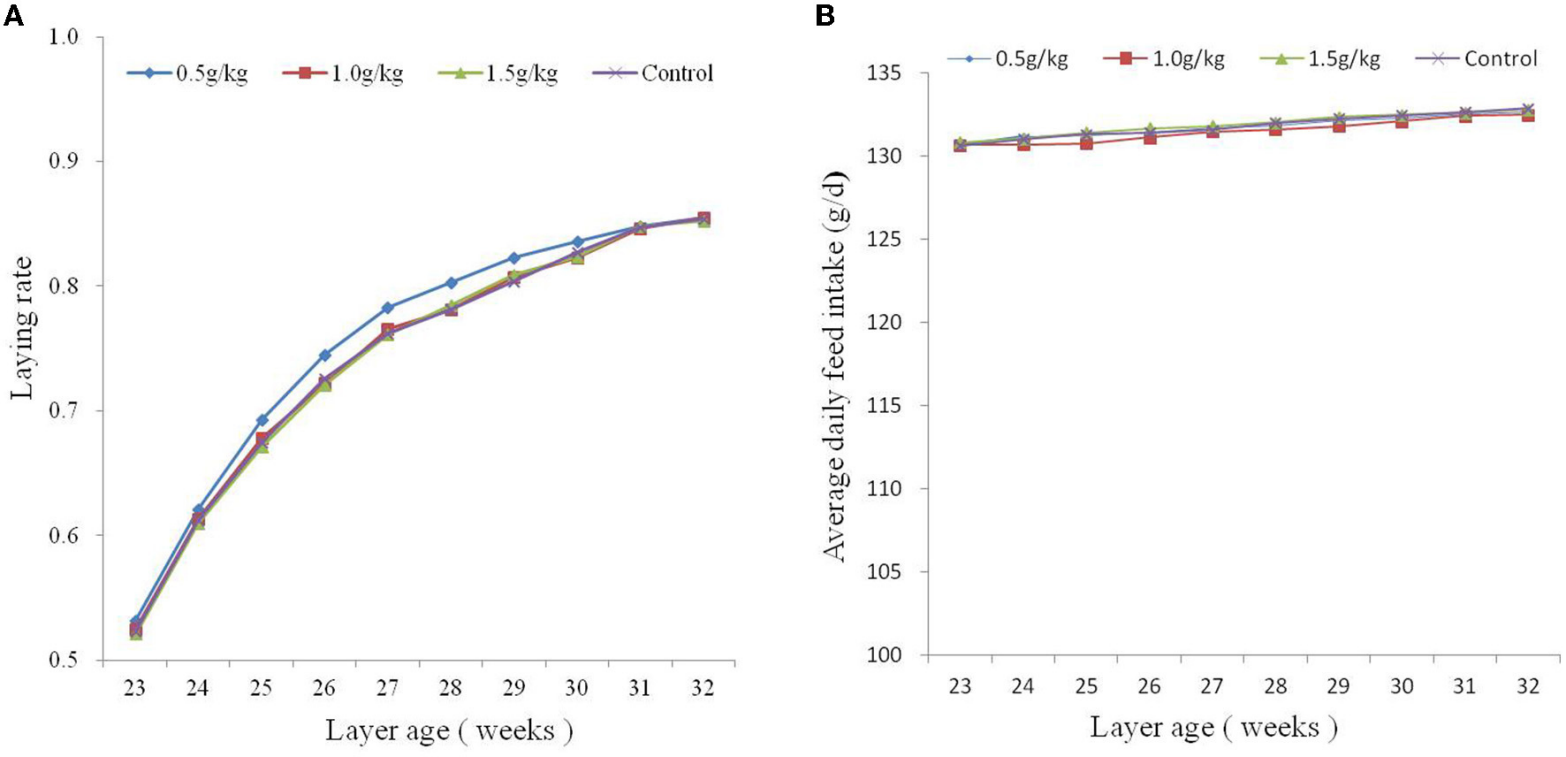
Plots of average daily feed intake **(A)** and laying rate **(B)** from 23 to 32 weeks of age.

The effect of dietary SBE on production performance is listed in Table 2, the addition of 1.0 g/kg SBE significantly increased (*P* < 0.05) the average egg weight and feed conversion ratio of laying ducks. However, the addition of SBE to the diet had no significant (*P* > 0.05) effect on the average daily feed intake and laying rate of laying ducks. Although the difference in egg production rate was not significant, it can be found from Figure 1A that feeding 0.5 g/kg of SBE had a tendency to increase egg production rate in the middle part of the experiment, indicating that SBE has some effect in increasing egg production rate of ducks. Combined with the Figure 1B, the average daily feed intake was generally not very different between groups and the trend was not obvious.

**Table 2:** Effect of dietary SBE on production performance of laying ducks^a^.

**Items^b^**	**SBE add levels, g/kg**				**SEM**	***P*-value**
	**Control**	**0.5**	**1.0**	**1.5**		
ADFI, g/d	131.850	131.857	131.478	131.991	0.085	0.927
AEW, g	61.891^b^	63.743^ab^	64.110^a^	61.391^b^	0.402	0.033
LR	0.7373	0.7504	0.7385	0.7349	0.008	0.927
FCR	2.718^a^	2.585^b^	2.501^bc^	2.710^ab^	0.017	0.044

a Different letters within a row denote significant differences (P < 0.05).

b Values represent the mean of six replicates (n = 30).

c ADFI, average daily feed intake; AEW, average egg weight; LR, laying rate; FCR, feed conversion ratio.

### 3.2. Serum biochemical indices

Effect of dietary SBE on serum biochemical indexes is presented in Table 3, compared to the control group the addition of 1.0 and 1.5 g/kg SBE significantly reduced (*P* < 0.05) the serum TC and TG levels, while serum GSH-Px and SOD levels were significantly higher (*P* < 0.05), and 1.5 g/kg SBE group significantly reduced (*P* < 0.05) levels of serum LDL-C. However, 0.5 g/kg SBE group significantly increased (*P* < 0.05) levels of serum T-AOC, IgG, IgA, and IgM than control group. No significant (*P* > 0.05) effect on the levels of HDL-C and MDA were detected among all groups.

**Table 3:** Effect of dietary SBE on serum biochemical indexes of laying ducks^a^.

**Items^b^**	**SBE add levels, g/kg**				**SEM**	***P*-value**
	**Control**	**0.5**	**1.0**	**1.5**		
**Serum lipid index**						
TC, mmol/L	4.074^a^	3.434^ab^	3.235^b^	3.301^b^	0.144	0.014
TG, mmol/L	4.040^a^	3.126^ab^	2.161^b^	2.762^b^	0.226	0.019
HDL-C, mmol/L	3.208	3.520	3.975	3.353	0.122	0.181
LDL-C, mmol/L	5.824^a^	5.313^ab^	4.893^ab^	3.787^b^	0.331	0.001
**Serum antioxidant index**						
SOD, U/mL	81.781^b^	89.478^a^	88.597^a^	85.619^ab^	0.936	0.046
T-AOC, U/mL	2.282^b^	2.544^b^	3.679^a^	3.402^a^	0.157	0.002
MDA, nmol/mL	8.824	10.441	9.471	8.721	0.518	0.546
GSH-Px, U/L	136.559^b^	155.544^a^	147.401^ab^	140.396^b^	2.502	0.043
**Serum immunity index**						
IgG, g/L	1.807^b^	2.342^a^	1.543^c^	1.618^bc^	0.065	< 0.001
IgA, g/L	0.220^b^	0.251^a^	0.158^d^	0.185^c^	0.007	< 0.001
IgM, g/L	0.384^b^	0.537^a^	0.283^c^	0.238^d^	0.019	< 0.001

a Different letters within a row denote significant differences (P < 0.05).

b Values represent the mean of six replicates (n = 12).

c TC, total cholesterol; TG, triglyceride; HDL-C, high-density lipoprotein cholesterol; LDL-C, low-density lipoprotein cholesterol; SOD, superoxide dismutase; T-AOC, total antioxidant capacity; MDA, malondialdehyde; GSH-Px, glutathione catalase; IgG, immunoglobulin G; IgA, immunoglobulin A; IgM, immunoglobulin M.

### 3.3. Egg quality

Effect of dietary SBE on egg quality is given in Table 4, Compared to the control group, supplementation with 0.5 g/kg SBE to the diet significantly increased (*P* < 0.05) the egg shell strength of duck eggs, adding of 0.5 and 1.0 g/kg SBE significantly increased (*P* < 0.05) the yolk weight of duck eggs, and 1.5 g/kg SBE group significantly increased (*P* < 0.05) the egg white height and haugh unit of duck eggs. In addition, feeding 1.0 and 1.5 g/kg SBE significantly reduced (*P* < 0.05) yolk cholesterol. However, SBE did not affect (*P* > 0.05) the egg shape index, shell thickness, yolk color and yolk specific gravity of duck eggs in comparison with the control group.

**Table 4:** Effect of dietary SBE on egg quality of laying ducks^a^.

**Items^b^**	**SBE add levels, g/kg**				**SEM**	***P*-value**
	**Control**	**0.5**	**1.0**	**1.5**		
Average egg weight, g	64.661^b^	65.154^b^	68.383^a^	64.476^b^	0.356	< 0.001
Egg-shaped index	1.357	1.368	1.361	1.351	0.006	0.789
Eggshell strength, kgf/cm^2^	45.517^b^	53.419^a^	48.506^ab^	47.489^b^	0.988	0.037
Eggshell thickness, mm	0.231	0.234	0.239	0.231	0.002	0.515
Yolk weight g	19.001^c^	20.544^ab^	20.846^a^	19.714^bc^	0.182	0.001
Yolk specific gravity	0.300	0.309	0.310	0.303	0.002	0.090
Albumen height, mm	6.293^b^	6.444^ab^	6.473^ab^	6.867^a^	0.103	0.036
Yolk color	11.278	11.222	11.444	10.611	0.140	0.167
Haugh unit	75.993^b^	76.750^b^	77.479^ab^	80.571^a^	0.613	0.041
Yolk cholesterol, mmol/gprot	0.516^a^	0.459^ab^	0.416^b^	0.444^b^	0.012	0.026

a Different letters within a row denote significant differences (P < 0.05).

b Values represent the mean of six replicates (n = 18).

### 3.4. Amino acids in eggs

The effect of addition of SBE to the diet on amino acids in eggs is listed in Table 5. The inclusion of SBE significantly increased (*P* < 0.05) the content of essential amino acids and total amino acids compared with the control group, while the content of non-essential amino acids in eggs was significantly increased (*P* < 0.05) when adding of 0.5 and 1.0 g/kg SBE. In addition, SBE significantly increased (*P* < 0.05) the contents of threonine, serine, leucine, phenylalanine, histidine, lysine, aspartic acid, and methionine in eggs. Interestingly, the addition of 0.5 and 1.0 g/kg of SBE significantly increased (*P* < 0.05) the content of glutamic acid and tyrosine in the fresh tasting amino acids of eggs. SBE did not affect (*P* > 0.05) the content of arginine in eggs.

**Table 5:** Effect of dietary SBE on amino acids n eggs^a^.

**Items, %^b^**	**SBE add levels, g/kg**				**SEM**	***P*-value**
	**Control**	**0.5**	**1.0**	**1.5**		
Asparagine^*^	3.852^b^	4.088^a^	4.176^a^	4.040^a^	0.040	0.005
Threonine	2.450^b^	2.587^a^	2.629^a^	2.619^a^	0.026	0.019
Serine	3.328^b^	3.464^a^	3.494^a^	3.444^a^	0.024	0.032
Glutamic acid^*^	5.786^b^	6.197^a^	6.192^a^	5.803^b^	0.065	0.001
Glycine^*^	1.431^b^	1.477^ab^	1.525^a^	1.510^a^	0.013	0.010
Alanine^*^	2.117^b^	2.127^b^	2.222^a^	2.176^ab^	0.015	0.025
Valine	2.640^c^	2.734^bc^	2.795^ab^	2.826^a^	0.024	0.004
Methionine	1.308^c^	1.728^a^	1.386^b^	1.674^a^	0.055	< 0.001
Isoleucine	1.982^c^	2.013^bc^	2.093^a^	2.067^ab^	0.015	0.012
Leucine	3.524^b^	3.665^a^	3.731^a^	3.695^a^	0.029	0.027
Tyrosine^*^	2.010^b^	2.190^a^	2.151^a^	2.075^b^	0.024	0.005
Phenylalanine^*^	3.182^b^	3.334^a^	3.390^a^	3.334^a^	0.028	0.015
Histidine	0.965^b^	1.009^a^	1.030^a^	1.013^a^	0.009	0.025
Lysine	3.078^b^	3.214^a^	3.286^a^	3.237^a^	0.027	0.011
Argnine	2.371	2.428	2.476	2.461	0.016	0.089
Proline	1.634^b^	1.670^b^	1.755^a^	1.718^ab^	0.009	0.009
TAA	41.660^b^	43.926^a^	44.331^a^	43.693^a^	0.363	0.011
EAA	19.130^b^	20.284^a^	20.341^a^	20.465^a^	0.185	0.007
NEAA	22.530^c^	23.642^ab^	23.990^a^	23.228^bc^	0.192	0.011
UAA	18.379^c^	19.414^ab^	19.656^a^	18.939^bc^	0.168	0.006
EAA/TAA	45.92	46.18	45.88	46.84		
EAA/NEAA	84.91	85.80	84.79	88.10		

a Different letters within a row denote significant differences (P < 0.05).

b Values represent the mean of six replicates (n = 3).

c TAA, total amino acids; EAA, essential amino acids; UAA, umami amino acids; EAA, threonine + valine + methionine + isoleucine + leucine + phenylalanine + lysine; UAA, asparagine + glutamic acid + glycine + alanine + tyrosine + phenylalanine.

* Belong to umami amino acids.

### 3.5. Fatty acid profile in eggs

The effect of addition of SBE to the diet on fatty acids in eggs is listed in Table 6. Compared to the control group, feeding of 1.0 g/kg SBE significantly reduced (*P* < 0.05) the content of saturated fatty acids (SFA) in eggs. In addition, duck eggs with SBE displayed significantly decrease (*P* < 0.05) in the contents of pentadecanoic acid (C15:0), heptadecanoic acid (C17:0), heneicosan ic acid (C21:0), elaidic acid (C18:ln9t), heptadecenoic acid (C17:1), linoleic acid (C18:2n6c), linolelaidic acid (C18:2n6t), eicosadienoic acid (C20:2), α-linolenic acid (ALA: C18:3n3), docosapentaenoic acid (DPA: C20:5), and eicosatrienoic acid (C20:3n6). However, the contents of total MUFA of eggs in SBE groups were higher than (*P* < 0.05) that in the control group. Among them, the contents of oleic acid (C18:ln9c) and gondoic acid (C20:ln9) in MUFA were significantly increased (*P* < 0.05). Although SBE reduced the content of total polyunsaturated fatty acids (PUFA), but significantly increased (*P* < 0.05) the content of docosahexaenoic acid (DHA: C22:6n-3) when adding 1.0 and 1.5 g/kg SBE to the diets. In contrast, SBE had no obvious (*P* > 0.05) effect on the content of lauric (C12:0) and myristic (C14:0) acids in eggs.

**Table 6:** Effect of dietary SBE on fatty acids in eggs^a^.

**Items, %^b^**	**SBE add levels, g/kg**				**SEM**	***P*-value**
	**Control**	**0.5**	**1.0**	**1.5**		
**Saturated fatty acid**						
Lauric acid, C12:0	0.019	0.016	0.017	0.016	0.0006	0.115
Myristic acid, C14:0	0.420	0.456	0.432	0.468	0.009	0.159
Myristoleate, C15:0	0.028^a^	0.022^b^	0.021^c^	0.020^c^	0.0009	< 0.001
Palmitate, C16:0	23.686^b^	23.577^b^	23.618^b^	24.203^a^	0.086	0.005
Margaric acid, C17:0	0.097^a^	0.069^b^	0.066^b^	0.057^c^	0.005	< 0.001
Stearic acid, C18:0	5.909^a^	5.849^a^	5.320^b^	5.267^b^	0.094	< 0.001
Arachidic acid, C20:0	0.036^a^	0.037^a^	0.032^b^	0.026^c^	0.001	< 0.001
Heneicosanoic acid, C21:0	0.016^b^	0.019^a^	0.019^a^	0.019^a^	0.0005	0.041
Behenic acid, C22:0	0.279^b^	0.303^a^	0.281^b^	0.284^b^	0.003	0.001
lignoceric acid, C24:0	0.041^a^	0.040^a^	0.033^b^	0.034^b^	0.001	< 0.001
**Monounsaturated fatty acid**						
Myristoleate, C14:1	0.034^b^	0.042^b^	0.041^b^	0.059^a^	0.003	0.001
Palmitoleate, C16:1	2.675^bc^	2.474^c^	2.704^b^	2.997^a^	0.067	0.001
Heptadecenoic acid, C17:1	0.078^a^	0.067^bc^	0.070^b^	0.065^c^	0.002	0.001
Elaidic acid, C18:ln9t	0.208^a^	0.075^c^	0.163^b^	0.137^b^	0.020	< 0.001
Oleic acid, C18:ln9c	55.165^c^	56.338^b^	57.082^a^	56.357^b^	0.225	0.001
Eicosenoic acid, C20:ln9	0.020^c^	0.235^a^	0.125^b^	0.105^b^	0.044	< 0.001
**Polyunsaturated fatty acid**						
Linolelaidic acid, C18:2n6t	0.083^a^	0.006^c^	0.056^b^	0.064^b^	0.011	< 0.001
Linoleic acid, C18:2n6c	9.162^a^	8.322^b^	7.834^bc^	7.585^c^	0.197	0.001
Linolenic acid methyl ester, C18:3n3 (ALA)	0.370^a^	0.105^c^	0.216^b^	0.193^b^	0.044	< 0.001
Methyl linolenate, C18:3n6	0.147^b^	0.145^b^	0.165^a^	0.164^a^	0.003	< 0.001
Eicosadienoic acid, C20:2	0.190^a^	0.184^b^	0.174^c^	0.142^d^	0.006	< 0.001
Eicosatrienoic acid, C20:3n6	0.211^a^	0.136^bc^	0.118^c^	0.164^b^	0.021	< 0.001
Arachidonic acid, C20:4n6	1.357^a^	1.261^b^	1.152^c^	1.307^ab^	0.025	0.001
Diphenylamine, C22:5n3 (DPA)	0.150^a^	0.103^b^	0.104^b^	0.097^b^	0.006	< 0.001
Docosahexaenoic Acid, C22:6n3 (DHA)	0.037^b^	0.036^b^	0.089^a^	0.090^a^	0.012	< 0.001
Total SFAs	32.941^b^	32.773^b^	32.458^c^	33.319^a^	0.102	0.001
Total MUFAs	63.729^b^	65.005^a^	65.343^a^	64.851^a^	0.105	0.003
Total PUFAs	9.435^a^	8.513^b^	8.064^bc^	7.790^c^	0.036	0.001
Total n-3 PUFAs	0.557^a^	0.245^c^	0.410^b^	0.379^b^	0.053	< 0.001
Total n-6 PUFAs	10.961^a^	9.871^b^	9.325^b^	9.283^b^	0.020	0.001

a Different letters within a row denote significant differences (*P* < 0.05).

b Values represent the mean of six replicates (*n* = 3).

c Among them, n-3 PUFA includes C18:3n3, C22:6n3 and DPA; n-6 PUFA includes C18:2n6c C18:2n6t C18:3n6, C20:3n6 and C20:4n6.

## 4. Discussion

In laying duck farming production, economic efficiency is usually increased by increasing the average egg weight or improving the feed conversion ratio. Egg white is one of the main factors affecting the weight of eggs. Egg white are reported to consist of ovalbumin and oval mucin, and are secreted and synthesized in the enlarged portion of the oviduct (also known as the protein-secreting portion) (23). The synthesis and secretion of ovalbumin is regulated by estrogens (24). Among them, yolk protein is the main protein in egg yolk, and estrogen induces the synthesis of yolk protein (25). It has been reported that quercetin in flavonoids can increase the synthesis of estrogen (26), thus regulate yolk protein synthesis, increasing yolk and egg white weights, and it is noteworthy that the increased yolk weight in this study was an important cause of the increased egg weight. Chand et al. (27) confirmed that the addition of sea buckthorn seeds to the ration increased the weight of eggs. However, SBE does not consistently increase egg weight, and one study showed that estrogen promotes calcium absorption (28), but excessive flavonoids inhibit estrogen production (29), therefore, the lack of calcium leads to egg weight loss. There was no significant difference in average daily feed intake between the groups in this study, however, the increase in egg weight led to a decrease in feed conversion ratio, this was confirmed by the findings of BenMahmoud et al. (10).

TG in egg yolk is synthesized by the liver, transported to the ovary *via* the bloodstream, and absorbed into the developing follicle *via* receptor-mediated endocytosis (4). Studies have shown that flavonoids can downregulate several adipogenic gene transcription factors, thereby reducing TG levels (30), and Yang et al. (31) showed that SBF can reduce serum TG levels. The source of TC is mainly through two routes: *in vivo* synthesis and dietary intake, with *in vivo* synthesis being mainly by the liver and, to a lesser extent, by the ovaries. Dietary intake is obtained through food. In addition to the TC required for the maintenance of the body, the remaining 2/3 of the TC in female birds is transported by the carrier LDL-C through the blood to the ovary, where it enters the follicle through receptor-mediated endocytosis, is deposited in the yolk, and is finally excreted by egg laying (30). During the synthesis of TC, HMG-CoA (3-hydroxy-3-methylglutaryl-coenzyme A) reductase serves as a key rate-limiting enzyme in the TC synthesis pathway, and SBF inhibits the synthesis of HMG-CoA reductase, thereby inhibiting TC synthesis (32). Some studies have confirmed that consumption of sea buckthorn fruit flavonoids can reduce blood TG and TC levels (18). In the current study, addition of 1.5 g/kg SBE, reduced serum LDL-C levels, which is consistent with the results of Krejcarová et al. (33) and Ma et al. (34), confirming the ability of SBE to reduce lipids. This indicates that the addition of SBE to the diet can reduce cholesterol deposition in Egg.

Studies have demonstrated that quercetin can reduce oxidative stress in follicular granulosa cells and ensure normal ovarian development (35). Moreover, antioxidants can delay ovarian decline and increase the useful life of laying hens (36). The metabolism of the body is accompanied by an oxidative process that generates free radicals along with the formation of reactive oxygen species (ROS) and reactive nitrogen species (RNS) that are harmful to the body, such as superoxide and hydrogen peroxide, and the body maintains the oxidative and antioxidant balance by scavenging free radicals (37). And the antioxidant effect is by enhancing the activity of antioxidant enzymes and inhibiting the activity of related oxidative enzymes, SOD and GSH-Px among antioxidant enzymes can induce the production of ROS scavenging enzymes. Studies have shown that SBF regulates Peroxidase through the Nrf_2_-ARE (nuclear related factor 2-antioxidant response element) signaling pathway, and SOD is responsible for the breakdown of superoxide anions into H_2_O_2_ and O_2_. GSH-Px further reduces the active peroxide to harmless alcohol and water (38). T-AOC reflects the body's ability to resist oxidation. In the present study, SBE could increase the content of SOD, GSH-Px, and T-AOC in serum, which indicated that SBE may improve the anti-oxidation ability of laying ducks and slow down the damage of oxidative stress. However, the content of SOD and GSH-Px in serum were decreased with further addition of SBE, indicating that excessive SBE could not further improve the antioxidant capacity of laying ducks.

The immunity of the organism is related to immune factors. Sea buckthorn fruit flavonoids improve the immunity of the organism by modulating immune-related regulatory factors *in vitro* and stimulating pro-inflammatory factors (IL-6, interleukin-6) and tumor necrosis factor (TNF-α, tumor necrosis factor-α) (39). The intestine is the largest immune organ of the animal organism (40). Attri et al. (41) found that sea buckthorn juice increased the diversity of Lactobacillus and Bacteroides in the colonic site, and a substantial increase in probiotics such as Bifidobacterium was found in the descending colonic site, and Bifidobacterium inhibited harmful bacteria, improve gastrointestinal barrier function, maintain intestinal microecological stability, and regulate intestinal immune homeostasis (42). Bifidobacteria can also promote the growth of B lymphocytes and regulate immune function (43). Organismal immunoglobulins are mainly composed of IgG, IgA, and IgM and are synthesized and secreted by B lymphocytes (44). It can be speculated that SBE may promote the growth of B lymphocytes by increasing the number of intestinal bifidobacteria, thereby increasing the content of IgG, IgA, and IgM in serum immunoglobulins. In this study, the serum IgG, IgA, and IgM contents of ducks were significant at 0.5 g/kg SBE addition, but with the increase of SBE addition, the IgG, IgA, and IgM contents decreased instead, probably because the high SBE addition changed the intestinal microbial structure of ducks in a direction unfavorable to the improvement of immunity, and even decreased immunity. In addition, methionine and cysteine have the effect of enhancing immune function (45), and the addition of SBE in this study increased the content of methionine and cysteine in eggs, which in turn improved the immunity of laying ducks.

Eggshell strength, egg white height, and haugh units are important indicators for evaluating egg quality. Calcium has a great influence on eggshell indicators, and increasing calcium absorption can improve eggshell strength and eggshell thickness (46). Estrogen has been shown to promote calcium absorption (28), the intestine and kidney contain a large number of estrogen receptors (47). When flavonoids bind to estrogen receptors on the small intestine and kidney, they promote calcium absorption in the small intestine and calcium reabsorption in the kidney. Flavonoids can improve eggshell strength by modulating estrogen and thus calcium metabolism (48). In the current study, supplementation with SBF to the ration could improve the eggshell strength of duck eggs, excess flavonoids inhibited estrogen synthesis (29), were responsible for the decrease in eggshell strength. A previous study showed that antioxidant properties are critical to maintaining the antioxidant protection of the oviduct during eggshell formation (49), so improving the antioxidant capacity is also an important reason for the improvement of the eggshell strength. Therefore, the amount of SBE added to the ration should not be too high in production. In addition SBF can also increase the synthesis and secretion of egg mucin by regulating estrogen (50), and β-ovalmucin in eggmucin combines with O-glycoside carbohydrates to form a gel structure that makes egg white sticky and directly increases egg white height (51). Egg white height was positively correlated with haugh unit, and increased egg white height in this study led to increased haugh units.

In addition to the apparent egg quality, the content and type of amino acids and fatty acids in duck eggs are also important indicators of egg quality. It has been shown that genistein flavonoids activate MAPK signaling pathway (activating Ser/Thr-protein kinase) and up-regulate insulin signaling pathway and glycolysis process in chicken liver to promote the conversion of glucose to amino acids (52), thus it was hypothesized that the addition of SBF to the diet could increase the TAA content in eggs. The higher the UAA content in the egg, the better the egg taste. When the expression of umami substance regulatory genes was high, the content of umami substance in meat also increased (53). In addition, antioxidants also reduced the loss of UAA, and it was speculated that SBE could regulate the expression of umami substance genes, which needed further verification. In the present study, the total UAA content in the eggs increased, especially the content of Asp, Glu, and Tyr increased significantly. The highest UAA content was found when 1.0 g/kg SBE was added, and the UAA content decreased when 1.5 g/kg SBE was added. Therefore, the addition of SBE could increase the content of EAA in duck eggs, and the moderate amount of SBE may increase the content of TAA, NEAA, and UAA in duck eggs, while the excessive amount of SBE may result in the decrease of content of TAA, NEAA, and UAA in duck eggs.

Most fatty acids in birds are synthesized and metabolized by the liver (54) and deposited in eggs *via* blood transport. In this study, the levels of SFA, PUFA, n-3 PUFA, and n-6 PUFA were reduced in eggs. On the one hand, peroxisome proliferator-activated receptor-alpha (PPARα) are important transcription factors for hepatic fatty acid metabolism (55), SBF can up-regulate the expression of PPARα (56), promote fatty acid oxidation and inhibit fatty acid synthesis (57), thus reducing fatty acid content (58). Additionally, flavonoids inhibit fatty acid synthase (FAS) activity to reduce fatty acid synthesis (59), thus suggesting that SBE reduces fatty acid content in eggs. Furthermore, fatty acids are the main component of TG (60), and in this study, SBF reduced serum TG levels, which resulted in lower fatty acid levels in eggs. In the present study SBE reduced the content of ALA and DPA in eggs, but interestingly inclusion of 1.0 and 1.5 g/kg SBE increased the content of DHA in eggs, which was 2.4 times higher than the control group. It has been shown that the human body can convert ALA to DHA by prolonging enzymes and desaturases (3), and DPA in laying hens can be efficiently converted to DHA (61). It is speculated that SBE may promote the conversion of ALA and DPA to DHA in laying ducks.

## 5. Conclusion

The addition of 1.0 g/kg SBE to the diet may increase the egg weight of laying ducks, improve the utilization of feed nutrients. Appropriate addition of SBE to the diet can improve the quality of eggs and the content of amino acids in eggs. SBE could also reduce blood lipids and yolk cholesterol, improve the fatty acid profile of eggs, and increase the antioxidant capacity and immunity of laying ducks. Therefore, these results suggest that SBE can be used as an effective feed additive in laying duck production.

## Data availability statement

The original contributions presented in the study are included in the article/supplementary material, further inquiries can be directed to the corresponding author.

## Ethics statement

The animal study was reviewed and approved by Guizhou University Sub-committee of Experimental Animal Ethics (Guiyang, China; No. EAE-GZU-2021-E012).

## Author contributions

B-nY: data collation and draft writing. S-lY: revise the first draft and supervise the completion of the test. F-yL, J-yY, AL, B-gZ, and GF: assist with feeding trials and writing. All authors have read and approved the final manuscript.
